# Therapeutic Implications of Estrogen for Cerebral Vasospasm and Delayed Cerebral Ischemia Induced by Aneurysmal Subarachnoid Hemorrhage

**DOI:** 10.1155/2014/727428

**Published:** 2014-03-02

**Authors:** Dale Ding, Robert M. Starke, Aaron S. Dumont, Gary K. Owens, David M. Hasan, Nohra Chalouhi, Ricky Medel, Chih-Lung Lin

**Affiliations:** ^1^Department of Neurosurgery, University of Virginia, Charlottesville, VA 22908, USA; ^2^Department of Neurosurgery, Tulane University, New Orleans, LA 70112, USA; ^3^Department of Molecular Physiology and Biophysics, Robert M. Berne Cardiovascular Research Center, University of Virginia, Charlottesville, VA 22908, USA; ^4^Department of Neurosurgery, University of Iowa, Iowa City, IA 52242, USA; ^5^Department of Neurological Surgery, Thomas Jefferson University, Philadelphia, PA 19106, USA; ^6^Department of Neurosurgery, Kaohsiung Medical University Hospital, No. 100, Tzyou 1st Road, Kaohsiung, Taiwan; ^7^Faculty of Medicine, College of Medicine, Kaohsiung Medical University, Kaohsiung 807, Taiwan

## Abstract

Cerebral vasospasm (CV) remains the leading cause of delayed morbidity and mortality following aneurysmal subarachnoid hemorrhage (SAH). However, increasing evidence supports etiologies of delayed cerebral ischemia (DCI) other than CV. Estrogen, specifically 17**β**-estradiol (E2), has potential therapeutic implications for ameliorating the delayed neurological deterioration which follows aneurysmal SAH. We review the causes of CV and DCI and examine the evidence for E2-mediated vasodilation and neuroprotection. E2 potentiates vasodilation by activating endothelial nitric oxide synthase (eNOS), preventing increased inducible NOS (iNOS) activity caused by SAH, and decreasing endothelin-1 production. E2 provides neuroprotection by increasing thioredoxin expression, decreasing c-Jun N-terminal kinase activity, increasing neuroglobin levels, preventing SAH-induced suppression of the Akt signaling pathway, and upregulating the expression of adenosine A2a receptor. The net effect of E2 modulation of these various effectors is the promotion of neuronal survival, inhibition of apoptosis, and decreased oxidative damage and inflammation. E2 is a potentially potent therapeutic tool for improving outcomes related to post-SAH CV and DCI. However, clinical evidence supporting its benefits remains lacking. Given the promising preclinical data available, further studies utilizing E2 for the treatment of patients with ruptured intracranial aneurysms appear warranted.

## 1. Introduction

Spontaneous subarachnoid hemorrhage (SAH) secondary to rupture of intracranial aneurysms represents a relatively small fraction of strokes (5%). However, the morbidity and mortality associated with aneurysm rupture remain very high despite advances in the diagnosis and treatment of aneurysmal SAH [1]. Cerebral vasospasm (CV) is the leading cause of delayed morbidity and mortality following aneurysmal subarachnoid hemorrhage (SAH). While radiographic CV is present in up to 70% of SAH patients, clinically symptomatic CV occurs in only 20–30% [2]. However, it is now evident that CV alone is inadequate to completely explain the delayed neurological dysfunction which occurs in the one to two weeks following the ictus of aneurysm rupture [3, 4].

After the age of 40, aneurysmal SAH is more common in females [5, 6]. The typical age of menopause, when serum estrogen levels decrease dramatically, is approximately 50 years [7]. It remains controversial whether the temporal relationship between decreased estrogen levels and increased incidence of aneurysm rupture in women is causative [8]. Despite extensive research on the effect of estrogen on aneurysm formation, progression, and rupture, the efficacy of estrogen for the treatment of SAH-induced CV has not been well investigated. With this deficiency in mind, we review the pathogenesis of aneurysmal SAH-induced CV, delayed cerebral ischemia (DCI), and the potential role of estrogen, specifically 17*β*-estradiol (E2), in combating these two interrelated but distinct cerebrovascular disease processes.

## 2. Pathogenesis of Aneurysmal Subarachnoid Hemorrhage-Induced Cerebral Vasospasm

### 2.1. Smooth Muscle Cell Contractile Mechanisms

In order to properly understand the molecular mechanisms of CV, we first briefly describe the manner by which smooth muscle cells (SMC) in the media of cerebral vasculature regulate contraction and relaxation [9]. The process of SMC contraction begins with opening of voltage-gated or ligand-gated calcium (Ca) channels allowing the entry of Ca from the extracellular space and from within the sarcoplasmic reticulum into the cytoplasm. The binding of cytoplasmic Ca to calmodulin (CaM), forming the Ca-CaM complex, can then activate the enzyme myosin light chain kinase (MLCK) to phosphorylate myosin. Phosphorylation of myosin allows it to bind to actin. At a cellular level, the coupling of myosin to actin results in SMC contraction which translates to vasoconstriction at the physiologic level.

### 2.2. Mechanisms of Cerebral Vasospasm Secondary to Endothelial Injury

Endothelial dysfunction is one of the primary contributing factors to CV following aneurysmal SAH. Endothelins (ET) are the most potent endogenous activators of vasoconstriction. ETs are produced by the endothelium and play a key role in maintaining vascular homeostasis. In the setting of SAH, the cerebrospinal fluid (CSF) levels of ET-1, the most common ET isoform, have been shown to be increased [10]. ET-1 binds to the endothelin receptor of which the two best characterized and most studied isoforms are ET_A_ and ET_B_ [11]. Binding of ET-1 to ET_A_ and ET_B_ results in vasoconstriction via pathways mediated by protein kinase C (PKC) [12]. PKC, which is activated by diacylglycerol (DAG) generated by phospholipase C and Ca generated from the opening of inositol triphosphate (IP3) gated Ca channels in the sarcoplasmic reticulum, promotes further Ca influx by opening cell surface Ca channels. In addition to Ca-dependent vasoconstriction, PKC also mediates Ca-independent vasoconstriction and vascular remodeling via mitogen-activated protein kinase (MAPK) [13].

Nitric oxide (NO), also known as endothelium-derived relaxing factor, is produced from arginine by the enzyme nitric oxide synthase (NOS) which exists in constitutively expressed isoforms, endothelial NOS (eNOS), neuronal NOS (nNOS), and the inducible isoform iNOS [14]. NO induces SMC relaxation increasing intracellular levels of the second messenger cyclic guanine monophosphate (cGMP) via activation of guanylate cyclase. The mechanisms by which elevated cGMP levels promote vasodilation include prevention of SMC depolarization by inhibition of Ca influx, facilitation of SMC hyperpolarization by activation of potassium (K) channels, and inhibition of SMC contraction by dephosphorylation of myosin by myosin light chain phosphatase (MLCP) which is activated by a cGMP-dependent kinase [15]. Decreased availability of NO secondary to hemoglobin-mediated destruction of nNOS and endogenous asymmetric dimethylarginine (ADMA) mediated inhibition of eNOS also contributes to the development of CV [16].

### 2.3. Mechanisms of Cerebral Vasospasm Secondary to Inflammation and Smooth Muscle Cell Injury

Acute subarachnoid hemorrhage generates the product oxyhemoglobin (oxy-Hb) which results in vasoconstriction via generation of reactive oxygen species (ROS) such as superoxide and hydrogen peroxide [17]. These ROS scavenge NO thereby preventing vasodilation. Upregulation of Ca channels by oxy-Hb may increase the sensitivity of the cerebral vasculature to contractile stimuli and both prolong and potentiate vasoconstriction [18]. Furthermore, oxy-Hb also results in an inflammatory cascade in the walls of the cerebral vessels. Inflammation activates iNOS which, in contrast to its constitutively active counterparts, causes cellular damage by generation of NO in an oxidative environment which then reacts with free radicals to propagate ROS formation. The elevation of ROS levels by iNOS results in further local vascular inflammation. Activation of the inflammatory cascade is induced at the ictus of SAH and contributes to the subsequent development of CV. In an experimental SAH model in primates, infiltration of inflammatory cells into the walls of cerebral vessels was shown to be the highest, one week following SAH induction, which correlated with the peak severity of angiographic vasospasm [19].

Proteins which promote cell-cell interactions have been shown to be upregulated during the inflammatory response in order to facilitate the recruitment, adhesion, and transmigration of leukocytes [20]. Intercellular adhesion molecule-1 (ICAM-1) is a ligand for the receptor lymphocyte function-associated antigen-1 (LFA-1) which is ubiquitously expressed not only on all T-cells but also on other immune cells such as neutrophils, macrophages, and B-cells. The coupling of ICAM-1 to LFA-1 is a crucial initial step to the recruitment of inflammatory cells to the vessel wall. Increased expression of ICAM-1 has been demonstrated in endothelial cells following exposure of the adventitia to blood [21]. Aihara et al. [22] measured the level and evaluated the time course of cytokine and cell adhesion molecule gene expression following induction of SAH in canines. They determined that the peak expression of interleukin-1 (IL-1), IL-6, IL-8, and ICAM-1 was seven days after SAH which correlated with maximal arterial narrowing on angiography. These results implicate a prolonged inflammatory response in CV with a potential correlation between the magnitude of inflammation and the severity of vasoconstriction.

In addition to ICAM-1, other cell adhesion molecules, such as vascular cell adhesion molecule-1 (VCAM-1) and E-selectin, have shown to be increased in the CSF of patients following aneurysm rupture [23]. Nissen et al. [24] studied the serum levels of multiple cell adhesion molecules in aneurysmal SAH patients with and without delayed ischemic neurological deficit (DIND) and did not find differences in the levels of ICAM-1, VCAM-1, platelet endothelial cell adhesion molecule-1 (PECAM-1), or E-selectin between the two cohorts. The levels of P-selectin and L-selectin were significantly higher and lower, respectively, in the patients who developed DIND. While there is clearly a link between inflammation and CV, it is clear that the use of CSF and serum biomarkers to assess this correlation is imperfect. Refinement of current approaches or development of new biomarker assays is necessary before these approaches can achieve widespread clinical applicability.

SMC contractility is not only mediated by SAH-induced variations in ET-1 and NO levels, but also by alteration in the electrochemical balance of ions such as Ca, sodium (Na), potassium (K), and chloride (Cl). SAH causes SMC depolarization by activation of Ca and Na channels and inactivation of K channels. Nimodipine, a Ca channel blocker (CCB), is the only pharmacologic agent which has been clinically proven to reduce delayed morbidity and mortality from aneurysmal SAH, although it does not reduce the incidence of angiographic CV [25–27]. The clinical benefit of endovascular administration of intra-arterial CCBs, such as verapamil, is currently equivocal [28]. The K channel activator cromakalim has been shown, in an *in vivo *rabbit SAH model, to ameliorate vasospasm [29]. Pathological alterations in ion channel physiology are crucial mechanisms underlying the molecular and clinical manifestations of CV. However, artificial manipulation of ion balance alone is inadequate to prevent or reverse the disease process.

Additional evidence suggests that SMC contractile tone and the mechanisms which regulate SMC contraction may change over the time course of CV [30]. This suggests that changes in SMC physiology may be induced by SAH. While the role of SMC phenotypic modulation in aneurysm formation, progression, and rupture has been studied, its role in the inflammation associated with CV is currently unknown [31]. A recent study by Kim et al. [32] identified a single nucleotide polymorphism in the gene encoding the NaCl cotransporter SLC12A3. Despite extensive research into the molecular biology and biochemistry of aneurysmal SAH-induced CV, the mechanisms underlying its pathogenesis remain incompletely understood.

## 3. Pathogenesis of Aneurysmal Subarachnoid Hemorrhage-Induced Delayed Cerebral Ischemia Unrelated to Cerebral Vasospasm

DCI unrelated to CV is becoming increasingly recognized as a significant contributor to delayed morbidity in aneurysmal SAH patients. These non-CV sources of neurological dysfunction may provide an explanation for the continuing failures of clinical trials using pharmacological inhibitors which target CV [33, 34]. In addition to cerebral ischemia secondary to lack of blood supply in the setting of CV-induced vasoconstriction, global DCI occurs following SAH secondary to activation of proapoptotic pathways [35]. The initiation of proapoptotic mechanisms likely occurs with the acute brain injury which accompanies the ictus of SAH. The rapid increase in intracranial pressure cannot be compensated for by higher levels of cerebral blood flow thereby resulting in a decrease in cerebral perfusion pressure (CPP). This sudden drop in CPP activates the stress response transcriptional regulatory protein hypoxia-inducible factor 1*α* (HIF-1*α*) [36]. HIF-1*α* increases the production of BCL2/adenovirus E1B 19 kDa protein-interacting protein (BNIP3) which promotes apoptosis by releasing cytochrome c from the mitochondria. This subsequently activates downstream caspases and by sequestering the antiapoptotic protein Bcl-2.

In addition to modulation of apoptotic pathways, SAH also causes disruption of the blood-brain barrier (BBB) by activation of matrix metalloproteinases (MMPs) which degrade the vascular basement membrane [37, 38]. Park et al. [39] observed increased BBB permeability, increased cerebral edema, and apoptosis of endothelial cells and hippocampal and cortical neurons after SAH induction in rats. Administration of a pan-caspase inhibitor reduced BBB permeability, prevented development of cerebral edema, and improved neurological outcome. The integrity of the BBB is not only important in limiting the accumulation of cerebral edema, but it is also linked to the prevention of proinflammatory signals and neuronal apoptosis [40]. Another cerebrovascular alteration following SAH is the dysfunction of the microcirculation. Rather than vasoconstriction of large arteries, some studies have suggested that the narrowing of small parenchymal arterioles contributes to pathological alterations in regional cerebral blood flow and to the development of DCI [41, 42].

Microvascular platelet aggregation following SAH is another potential mechanism which may mediate DCI secondary to small vessel thrombosis and cortical and subcortical ischemia. Sehba et al. [43] detected microvascular platelet aggregation in SAH-induced rats by immunostaining for the glycoprotein IIb/IIIa (GPIIb/IIIa), the receptor on activated platelets responsible for mediating fibrin cross-linking. An autopsy study of 29 patients who died from aneurysm rupture identified a statistically significant correlation between the magnitude of microvascular thromboembolism burden, detected by immunostaining, and the histologic evidence of ischemia at autopsy as well as clinical evidence of DCI prior to death [44].

In addition to microcirculatory disease, widespread cortical depression may predispose SAH patients to DCI. Dreier et al. [45] performed electrocorticography on patients who were surgically treated for ruptured aneurysms and found spreading depolarizations in 72%. The electrocorticographic measurement of recurrent spreading depolarizations had 86% and 100% positive and negative predictive values, respectively, for the development of delayed ischemic neurological deficits. The authors proposed that repeated spreading depolarizations with prolonged depressions could predict the subsequent occurrence of DCI. It is likely that many of the aforementioned pathological mechanisms are interrelated with the development, propagation, and worsening of CV [46]. However, past clinical outcomes from aneurysmal SAH studies have taught us that the reversal of angiographic CV alone is inadequate to ameliorate the delayed morbidity and mortality associated with the rupture of an intracranial aneurysm.

## 4. Role of Estrogen in the Treatment of Cerebral Vasospasm

### 4.1. Estrogen Physiology

E2 is the most potent endogenous estrogen. Like other steroid hormones, E2 is derived from cholesterol. Cholesterol is initially converted to the intermediate progesterone products, pregnenolone and 17*α*-hydroxypregnenolone, which are then converted to the androgen intermediates, dehydroepiandrosterone, androstenediol, androstenedione, and testosterone. The androgen testosterone is then converted by the final enzyme in the synthetic pathway, aromatase, into E2. In a parallel pathway, androstenedione is converted by aromatase into estrone (E1) which, *in vivo*, is interconvertible with E2 [47]. E2 passes through the cell membrane to bind to the two isoforms of the estrogen receptor (ER), ER*α* and ER*β*, in the cytoplasm. The E2-ER complex then enters the cell nucleus to regulate the transcription of multiple genes [48]. Pharmacologic blockade of the physiologic effects of E2 targets the synthetic pathway or the receptor. E2 synthesis is decreased with gonadotropin-releasing hormone (GnRH) agonists (e.g., leuprolide, goserelin) and aromatase inhibitors (e.g., anastrozole, exemestane). Antagonists of the ER are more properly termed selective estrogen receptor modulators (SERM) since, rather than being pure antagonists, they are simultaneously partial agonists as well as antagonists [49]. The action of SERMs is tissue specific and varies depending on the relative ratio of coactivator to corepressors and on the conformation of the ER.

### 4.2. Effect of Estrogen on the Vasculature during Cerebral Vasospasm

E2 is a powerful vasodilator with the potential to prevent or reverse the vasoconstriction which occurs in CV. *In vitro* studies have demonstrated that E2 binding to ER*α* results in activation of eNOS through MAPK-dependent pathways [50]. *In vivo* evidence from continuous E2 treatment of SAH-induced animals showed attenuation of CV, decreased SAH-induced iNOS expression, and normal eNOS expression [51]. This implicates a dual role of E2 in the prevention of SAH-induced iNOS upregulation and the maintenance of normal eNOS activity (which is typically suppressed in the setting of SAH). Mechanistic data from *in vitro *studies by Zancan et al. [52] demonstrated abrogation of cytokine-induced iNOS upregulation by E2 treatment in cultured rat aortic SMCs. Blockade of E2 signaling with an ER*α* antagonist resulted in the absence of E2 modulation of iNOS expression.

**Figure 1:**
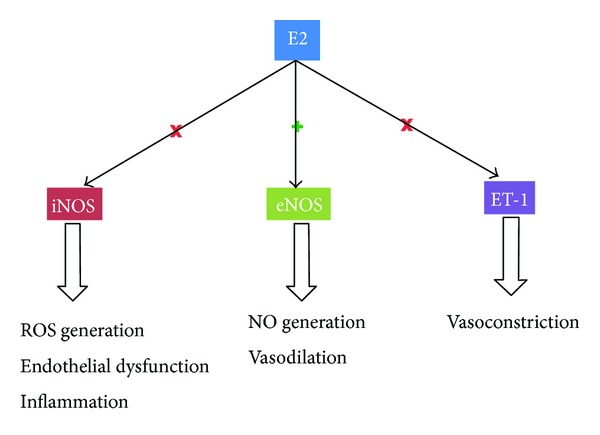
Pathways of E2-mediated facilitation of vasodilation and inhibition of vasoconstriction. E2: 17*β*-estradiol, eNOS: endothelial nitric oxide synthase, ET-1: endothelin-1, iNOS: inducible nitric oxide synthase, NO: nitric oxide, and ROS: reactive oxygen species.

Shih et al. [53] treated SAH-induced rats with E2 and a nonselective ER antagonist and found that E2 prevented post-SAH elevation of iNOS levels and CV in an ER-dependent mechanism. The study also examined the levels of p65, a subunit of nuclear factor *κ* light chain enhancer of activated B cells (NF*κ*B) and identified increased association of p65 and ER following administration of E2. The nuclear translocation of p65 was unaffected by E2 treatment. Therefore, a potential mechanism of E2-mediated vasodilation is the cytoplasmic sequestration of the transcription factor NF*κ*B by ER which prevents the NF*κ*B-dependent upregulation of iNOS instigated by aneurysmal SAH. Studies in extracranial vasculature have suggested that E2 may potentiate the effect of MMPs on the modulation of ET-mediated vasoconstriction [54]. Additional *in vivo* data from an experimental SAH model in rats demonstrated significantly decreased levels of ET-1 production in the cohort treated with E2 [55]. The ET-1 levels of the SAH animals treated with E2 were not significantly different from ET-1 levels of control animals. The mechanisms by which E2 mediates vasodilation are depicted in Figure 1.

## 5. Neuroprotective Mechanisms of Estrogen in the Setting of Aneurysmal Subarachnoid Hemorrhage

Evidence suggests that E2 may have neuroprotective properties [56]. E2 appears to diminish the risk of ischemic stroke and neurodegenerative disorders, such as Alzheimer's and Parkinson's disease [57]. Not all neuroprotection afforded by E2 is mediated by ER. Early studies found E2 to have antioxidant effects, via scavenging of ROS, which were unaffected by tamoxifen treatment [58]. Lee et al. [59] showed that E2 increased expression of the antioxidant thioredoxin (Trx) in a cGMP-dependent manner. Trx was demonstrated, in the same study, to abrogate lipid peroxidation, caspase-3 activation, and apoptosis in response to oxidative stress. Srivastava et al. [60] found that E2 decreased expression of the critical proinflammatory cytokine tumor necrosis factor *α* (TNF*α*) by causing reduced activity of c-Jun N-terminal kinase (JNK). Depression of JNK results in decreased phosphorylation of its downstream targets which heterodimerize to form the transcription factor activator protein-1 (AP-1). AP-1 transactivates TNF*α* by binding to its promoter region. Thus, E2-mediated disruption of AP-1 formation decreased transcription of TNF*α*. Xing et al. [61] subsequently demonstrated that the anti-inflammatory actions of E2 were dependent on the ER*β* receptor isoform. In addition to decreasing TNF*α* expression, E2 binding to ER*β* also hindered neutrophil chemotaxis by decreasing expression of P-selectin, ICAM-1, VCAM-1, monocyte chemoattractant protein-1 (MCP-1), and cytokine-induced neutrophils chemoattractant-2*β* (CINC-2*β*).

Neuroglobin (Ngb) is a protein which regulates neuronal oxygen homeostasis by binding to oxygen with a higher affinity than hemoglobin [62]. While the precise mechanism of Ngb has yet to be delineated, it likely contributes to the protection of the brain from oxidative damage by ROS. De Marinis et al. [63] found that *in vitro* treatment of mouse hippocampal neurons with E2 resulted in a threefold increase in Ngb levels which was mediated by the ER*β* receptor. The ER*β*-mediated upregulation of Ngb expression was dependent on the p38 class of MAPKs. Additionally, E2 afforded protection against apoptosis induced by hydrogen peroxide. This protective effect was abrogated in Ngb-silenced cells. Hota et al. [64] provided *in vivo* data to support the antiapoptotic role of Ngb during the neuronal stress response to hypoxic stimuli. Ngb was shown to stabilize the transcription factors HIF-1*α* and nuclear factor erythroid 2-related factor 2 (Nrf2) and prevent mitochondrial release of the caspase-activating protein cytochrome c.

**Figure 2:**
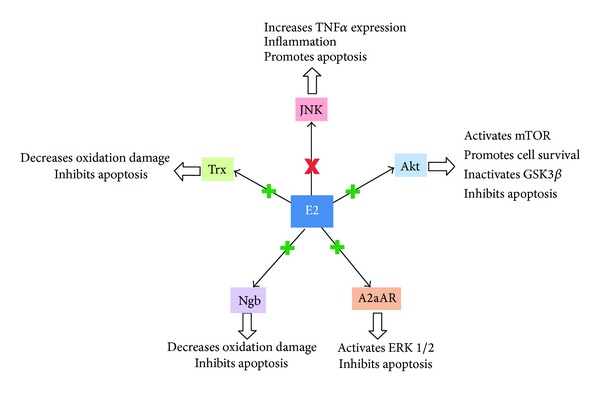
Pathways of E2-mediated neuroprotection. Akt: protein kinase B, E2: 17*β*-estradiol, ERK1/2: extracellular signal-regulated kinases 1 and 2, GSK3*β*: glycogen synthase kinase 3*β*, JNK: c-Jun N-terminal kinase, mTOR: mammalian target of rapamycin, Ngb: neuroglobin, TNF*α*: tumor necrosis factor *α*, and Trx: thioredoxin.

Recent *in vivo* evidence presented by Kao et al. [65] implicates the Akt signaling pathway in E2-mediated neuroprotection. Akt, otherwise termed protein kinase B (PKB), is downstream from phosphoinositide 3-kinase (PI3K) and upstream from the kinase known as mammalian target of rapamycin (mTOR). This complex signaling pathway involving the three kinases PI3K, Akt, and mTOR, integrates multiple inputs in order to promote cell growth and proliferation [66]. Downstream inactivation of glycogen synthase kinase 3*β* (GSK3*β*) by Akt is known to inhibit apoptosis [67]. Activation of ER*α* by E2 was shown to inhibit apoptosis in the dentate gyrus of rats afflicted with SAH by depressing the activity of the proapoptotic enzyme caspase-3 and by preventing the SAH-induced decrease in signaling through Akt [65]. Prior studies potentiating signaling through Akt-GSK3*β* pathway by other mechanisms have also shown attenuation of neuronal cell death in the setting of SAH [68, 69]. Furthermore, E2 has been found to exert antiapoptotic effects through upregulation of adenosine A2a receptor (A2aAR) and extracellular signal-regulated kinases 1 and 2 (ERK1/2) expression [70]. The activation of A2aAR and ERK1/2 results in inhibition of downstream apoptotic signaling pathways. The mechanisms by which E2 mediates neuroprotection are depicted in Figure 2.  Table 1 summarizes the molecular mechanisms underlying E2-mediated vasodilation and neuroprotection.

## 6. Limitations of Current Studies and Future Directions

Estrogen treatment is associated with multiple potential adverse effects which may dampen the enthusiasm for its use in the treatment of aneurysmal SAH patients. These risks include malignancies of the breast, endometrium, and ovaries, dysmenorrhea, gastrointestinal dysfunction, dyslipidemia, venous thrombosis, pulmonary embolism, myocardial infarction, and stroke [71–73]. Due to the relatively short clinical time course during which estrogen would be administered to treat CV and DCI (i.e., approximately two weeks), we believe the increased risk of estrogen-related cancers would be negligible. However, the potential systemic adverse effects of estrogen on patients, especially those with preexisting medical comorbidities, may be significant. It is possible that thromboembolic complications associated with estrogen therapy may offset any benefits afforded by its vasodilatory or neuroprotective mechanisms. While estrogen has been demonstrated to diminish CV and DCI in animal SAH models, it has yet to be tested in human trials for the treatment of patients with ruptured aneurysms. Therefore, the clinical safety profile and efficacy of estrogen have yet to be determined. Future studies are necessary to establish a dose-response relationship for estrogen in order to initiate early phase clinical trials.

## 7. Conclusions

Estrogen, specifically E2, possesses powerful vasodilatory, anti-inflammatory, and neuroprotective properties. Its current use for the treatment of CV remains limited to *in vivo* animal models of experimental SAH. It appears that the significant majority of E2-mediated neuroprotection occurs via ER*α*- and ER*β*-dependent mechanisms. The contribution of ER-independent mechanisms of E2 neuroprotection is relatively small. Therefore, successful pharmacologic modulation of ERs may provide a potential target of future clinical studies of E2 for the treatment of CV and DCI following aneurysmal SAH.

## Figures and Tables

**Table 1:** Summary of vasodilatory and neuroprotective mechanisms regulated by 17*β*-estradiol (E2).

Mediator	Physiology
Vasodilatory mechanisms regulated by E2	
Endothelial nitric oxide synthase (eNOS)	Constitutively expressed isoform of NOS which generates the vasodilatory mediator NO. E2 activates eNOS and prevents the SAH-induced decrease of eNOS function via MAPK-dependent pathways.
Inducible nitric oxide synthase (iNOS)	Inducible isoform of NOS expressed in stress responses (e.g., SAH) which contributes to the generation of reactive oxygen species. E2 abrogates SAH-induced iNOS expression by sequestering NF*κ*B, a transcriptional activator of iNOS.
Endothelin-1 (ET-1)	The most potent endogenous mediator of vasoconstriction. E2 decreases ET-1 production.

Neuroprotective mechanisms regulated by E2	
Thioredoxin (Trx)	Antioxidant enzyme which reduces oxidized proteins and diminishes stress-induced proapoptotic signaling. E2 increases Trx expression.
c-Jun N-terminal kinase (JNK)	JNK phosphorylates downstream proteins which heterodimerize to form AP-1, a transcriptional activator of TNF*α*. E2 decreases JNK activity.
Neuroglobin (Ngb)	Globin protein which binds to oxygen with a greater affinity than hemoglobin thereby regulating oxygen homeostasis in neurons. Ngb provides protection against ROS-induced oxidative damage and prevents apoptosis by stabilizing the transcription factors HIF-1*α* and Nrf2 and by inhibiting cytochrome c release from the mitochondria. E2 increases Ngb expression.
Protein kinase B (Akt)	Akt activates mTOR which promotes cell survival and inactivates GSK3*β* which promotes apoptosis. E2 prevents SAH-induced suppression of Akt.
Adenosine A2a receptor (A2aAR)	G-protein couple receptor which inhibits proapoptotic signaling pathways partially through activation of ERK 1/2. E2 increases expression of A2aAR and ERK 1/2.
